# Esc2 promotes telomere stability in response to DNA replication stress

**DOI:** 10.1093/nar/gkz158

**Published:** 2019-03-06

**Authors:** Signe W Jørgensen, Sascha E Liberti, Nicolai B Larsen, Michael Lisby, Hocine W Mankouri, Ian D Hickson

**Affiliations:** 1 Center for Chromosome Stability, Department of Cellular and Molecular Medicine, University of Copenhagen, Panum Institute, 2200 Copenhagen N, Denmark; 2 Center for Healthy Aging, Department of Cellular and Molecular Medicine, University of Copenhagen, Panum Institute, 2200 Copenhagen N, Denmark; 3 Department of Biology, University of Copenhagen, Ole Maaløes Vej, 2200 Copenhagen N, Denmark

## Abstract

Telomeric regions of the genome are inherently difficult-to-replicate due to their propensity to generate DNA secondary structures and form nucleoprotein complexes that can impede DNA replication fork progression. Precisely how cells respond to DNA replication stalling within a telomere remains poorly characterized, largely due to the methodological difficulties in analysing defined stalling events in molecular detail. Here, we utilized a site-specific DNA replication barrier mediated by the ‘Tus/*Ter*’ system to define the consequences of DNA replication perturbation within a single telomeric locus. Through molecular genetic analysis of this defined fork-stalling event, coupled with the use of a genome-wide genetic screen, we identified an important role for the SUMO-like domain protein, Esc2, in limiting genome rearrangements at a telomere. Moreover, we showed that these rearrangements are driven by the combined action of the Mph1 helicase and the homologous recombination machinery. Our findings demonstrate that chromosomal context influences cellular responses to a stalled replication fork and reveal protective factors that are required at telomeric loci to limit DNA replication stress-induced chromosomal instability.

## INTRODUCTION

Every time a cell divides, it must accurately duplicate all of its genetic material via the process of DNA replication. Errors or unscheduled delays in DNA replication can cause growth arrest, mitotic abnormalities and the acquisition of harmful mutations that potentially can promote cancer or premature aging (1,2). Certain regions of the genome are particularly challenging to replicate, including telomeres, the protective structures that cap the ends of all linear eukaryotic chromosomes. Telomeric regions have a propensity to form obstacles that can impede the progression of the DNA replication machinery (3). These obstacles include DNA secondary structures and repetitive elements, telomere-specific DNA-binding proteins, and active transcription of a non-coding RNA (TERRA; (4,5)). Disrupted fork progression at any locus generates so-called DNA replication stress, which in the case of a telomere can contribute to genomic rearrangements or loss of telomere sequences (telomere shortening) (6). Irreversible fork collapse in a telomere is particularly detrimental because the disrupted fork cannot be rescued by a second fork originating from an adjacent replication origin. Once telomeres become critically short or dysfunctional, they cause cells to cease dividing and enter a viable, non-dividing cellular state known as senescence (7). Because telomeres shorten as a result of genome duplication during cell proliferation, this is considered to be an effective anti-cancer mechanism that limits the number of times that a somatic cell can divide. However, the progressive accumulation of senescent cells within tissues is thought to contribute to the inevitable decline in tissue function during normal aging (8,9). Furthermore, cancer cells eventually reprogram their cellular networks to counteract telomere shortening and restore proliferative capacity (10). This occurs either through reactivation of telomerase (∼85% of cancers), or via the poorly characterized Alternative Lengthening of Telomeres (ALT) mechanism that requires the homologous recombination (HR) machinery (∼15% of cancers) (11,12). Therefore, stable telomere maintenance is inexorably linked to cancer and aging. However, much still remains to be defined about telomere replication and dynamics in normal dividing cells.

To better understand the molecular events occurring at a stalled replication fork, we developed an inducible, heterologous DNA replication barrier (the ‘Tus/*Ter* barrier’) that permits the detailed spatiotemporal analysis of site-specific replication fork stalling at a defined location (13,14). This replication fork barrier can be co-engineered with a *URA3* genetic reporter that allows detection of localized mutations and genome rearrangements that arise after the Tus/*Ter* barrier is activated. Previously, we used the Tus/*Ter* system to demonstrate that stalled replication forks can generate localized deletions and duplications at a non-telomeric locus (15). Furthermore, we demonstrated that Sgs1, a member of the evolutionarily conserved RecQ helicase family, counteracts the generation of these genome rearrangements. Of relevance to human pathology, mutations that inactivate either of two human RecQ helicases, BLM or WRN, cause Bloom’s syndrome and Werner’s syndrome, respectively, which are characterized by cancer predisposition, and premature aging (16).

In this study, we examined the consequences of site-specific replication fork stalling at a defined telomere. We demonstrate that replication fork stalling within a telomeric locus can promote chromosomal rearrangements that frequently lead to the loss of telomere-proximal DNA sequences. We show that the SUMO-like domain protein, Esc2, counteracts these genome rearrangements in telomeric regions through a mechanism that prevents aberrant, Mph1-driven, HR events. Our findings demonstrate that chromosomal context can lead to differential outcomes at a perturbed replication fork, and reveal proteins that operate at telomeric regions to limit chromosomal rearrangements following DNA replication stress.

## MATERIALS AND METHODS

### Strains and plasmids

The Tus-expression plasmids were described previously (17). All yeast strains used in this study (except for those used for high-throughput genetic screen) are isogenic derivatives of BY4741, which lack a 1.1 kb segment of the *URA3* gene on ChrV. The 21x*Ter* modules and Open reading frame (ORF) deletion cassettes were all integrated into the yeast genome by targeted HR (17).

### Growth conditions and flow cytometry

Yeast cultures were grown in Yeast extract peptone (YEP) medium (Formedium) supplemented with 3% sodium DL-lactate solution at 30°C. Cultures were synchronized in G1 with α-factor for 2.5 h (CASLO ApS). Tus expression was induced by adding 2% Galactose (final w/vol) during the G1-arrest. Release of cells from G1-arrest was achieved by centrifugation, washing and re-suspension of cells in fresh galactose-containing medium at 30°C. Cell-cycle stage was determined by flow cytometry using a Becton-Dickinson FACsCalibur and CellQuest software (BD Biosciences, Denmark).

### High-throughput genetic screen

21x*Ter* construct including *URA3* reporter was engineered into *TEL06R* of the MATα screen query strain as described above. Tus, under the control of the *GAL1* promoter, was engineered into the endogenous *URA3* locus on chromosome V of the same strain. The query strain was crossed with the MATa yeast gene deletion strains (18,19). Selection for diploids was carried out on solid YPD containing 300 mg/L hygromycin and 200 mg/L G418. Resulting diploids were sporulated on solid E-SPO containing 10 000 units/mL of penicillin and 10 000 µg/mL of streptomycin (Gibco by Life Technologies, cat. no. 15140-122). Haploids were selected for via multiple rounds of replica plating onto solid SC-Leu-Ura-Arg-Lys containing 50 mg/L canavanine, 50 mg/L thialysine, 300 mg/L hygromycin and 200 mg/L G418. MATa meiotic progeny containing the desired ORF deletion, *TEL06R*::URA3-21x*Ter* and *ura3*::GAL1-Tus-HPH were subjected to expression of Tus followed by selection for 5-FOA resistance (750 mg/L). Replica plating was done using the Rotor HDA from Springer Instruments. Following manual scoring, hits were re-tested in cells with either restrictive or permissive *Ter* sites. Validated hits were then constructed in BY4741 background for further analysis.

Genotypes of the library and query strains are as follows:

Library genotype: *MAT***a***orfΔ::kanMX4 his3Δ1 leu2Δ0 met15Δ0 ura3Δ0 TRP1 CAN1 LYS2 ADE2*
Query strain genotype: *MAT*α *can1Δ::STE2pr-LEU2 lyp1Δ his3Δ1 leu2Δ0 ura3::GAL1-Tus-HPH LYS2 MET15 TEL06R::URA3-21xTer*

### Analysis of mutation rates and types

Individual colonies picked from 2% raffinose (w/vol) plates were grown to saturation in non-selective medium containing 2% galactose (w/vol) at 25°C, and *URA3* and *CAN1* mutation rates were measured using fluctuation analysis (20,21). For WT and *esc2*, rates from all experiments have been combined to a single rate, which is used in all plots and for all statistical comparisons. Statistical analysis of differences in mutation rates between isogenic strains was performed with PRISM software, using a one-sided Mann–Whitney U test, and statistical significance was indicated when *P* < 0.01. For charts displaying ‘Tus-induced fold increase in 5-FOA resistance, (fold increase in 5-FOA resistance or fold increase in *URA3* mutation rate), the fold increase was determined for individual experiments, and bars represent values averaged across all experiments. Error bars represent the standard deviation, and statistical analysis of differences was performed using an unpaired *t*-test. Statistical significance was indicated when *P* < 0.05. For charts displaying ‘normalized *URA3* mutation rates’, the normalized *URA3* mutation rates (control rate subtracted from Tus rate) were determined for individual experiments, and bars represent values averaged across all experiments. Error bars represent the standard deviation, and statistical analysis of differences was performed using an unpaired *t*-test. Statistical significance was indicated when *P* < 0.05. For analysis of mutation types, cells grown on 2% raffinose plates were pinned onto non-selective plates containing 2% galactose. Plates were incubated at 25°C for 4 days, and then replica plated onto plates containing uracil and 5-flouro-orotic acid (5-FOA). Individual colonies were confirmed as being 5-FOA resistant before further analysis. For restriction digest analysis, DNA was extracted, digested with AfeI, SalI and EcoNI (from NEB), and analysed by 1-dimensional gel electrophoresis (1DGE). Restriction fragments were visualized by Southern blotting with probes spanning the EcoNI restriction site (Probe 1) or the SalI restriction site (Probe 2) as indicated in Figure 4A. Telomere tailing was performed using terminal transferase enzyme, as recommended by the supplier (NEB), before polymerase chain reaction with polyG and chromosome-specific primers. The *URA3* reporter was then sequenced using two different primers. Mutations were scored as events that were detectable in two sequence reads. Specific mutation rates were calculated by multiplying the fraction of a given mutation type with the mutation rate for the given strain.

### 2D gel analysis of DNA structures

Cell pellets were subjected to PUVA-crosslinking before DNA extraction. The hexadecyltrimethylammonium bromide method of DNA extraction was used, and 25 μg DNA was analysed by 2-dimension gel electrophoresis (2DGE), as described previously (17). DNA was digested with NheI or BspHI for visualization of *TEL06R* fragments and MfeI for *his2* fragments (as indicated in figure legends). All restriction enzymes were from NEB. *URA3* and/or *TEL06R*-specific probes were used for Southern blotting. QuantityOne software was used for quantification of signals in the 2D gels.

## RESULTS

### Replication fork stalling at a telomeric Tus/*Ter* barrier generates localized mutations

**Figure 1.**
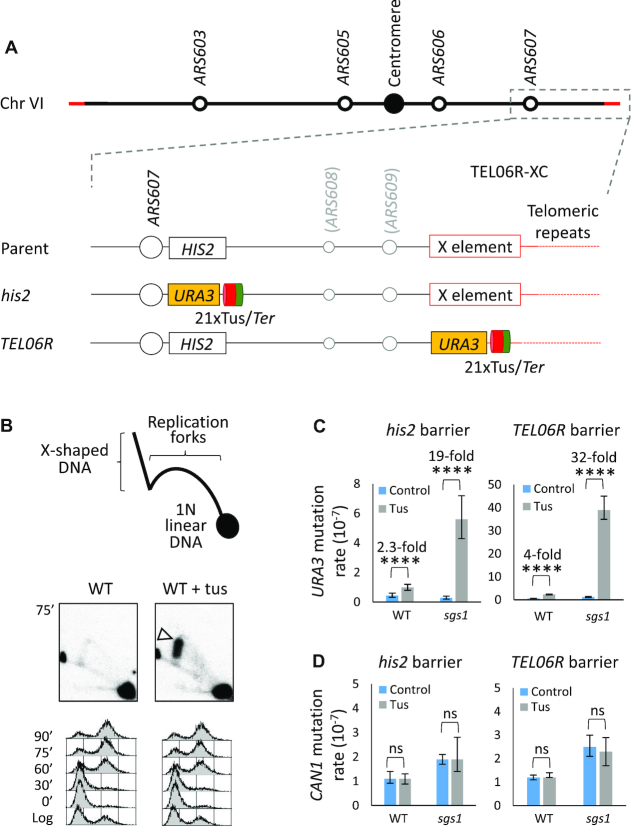
Site-specific replication fork stalling at Tus/*Ter* barriers causes localized mutagenesis. (**A**) Schematic diagram showing ChrVI and the position where the 21xTus/*Ter* barrier (incl. *URA3* reporter gene) was inserted at either *his2* or *TEL06R* (not drawn to scale). The red and green segments of Tus denote the restrictive and permissive faces, respectively. (**B**) Expression of Tus causes replication fork stalling at the *TEL06R* Tus/*Ter* barrier. Top panel depicts a diagram illustrating replication intermediates that can be visualized by 2DGE and Southern blotting. Genomic DNA was extracted at 75′ after G1 release, and BspHI-telomere fragments were analysed by 2DGE and Southern blotting using a *URA3-*specific probe. The white arrowhead indicates replication fork stalling at the Tus/*Ter* barrier in the cells expressing Tus. Cell cycle profiles are shown below. (**C**) *URA3* and (**D**) *CAN1* mutation rates were measured simultaneously for strains harbouring 21xTus/*Ter* at either *his2* or *TEL06R*. Error bars indicate 95% confidence limits, and the numerical values above the columns indicate the fold increase in mutation rate between isogenic strains with (grey) or without (blue) Tus expression. Statistical analysis of differences in mutation rates was performed using a one-sided Mann–Whitney U test, and significance is indicated only when *P* < 0.01 (**P < 0.01; ****P < 0.0001). ns = difference not statistically significant.

To investigate how chromosomal location influences the processing of a stalled replication fork, we inserted a *URA3*-21x*Ter* cassette into either of two defined locations on ChrVI (Figure 1A). To perturb DNA replication at a telomeric region, we engineered *URA3*-21x*Ter* into the sub-telomeric X-element of *TEL06R*. Most of the X-element (including the *ARS610* replication origin) was deleted, except for the terminal 56-bp. Because this locus is normally replicated at the final stages of S-phase, and there are no other telomere-proximal DNA replication origins beyond the Tus/*Ter* barrier that can rescue a stalled fork, this scenario is envisaged to be a particularly challenging type of site-specific DNA replication perturbation. Moreover, certain types of genomic rearrangements that would be lethal within chromosomal loci containing essential genes can potentially be recovered and analysed at a telomere. This is because of the lack of essential genes near the ChrVI right telomeric end, as well as the ability of telomerase to ‘heal’ a broken chromosome end through *de novo* telomere addition (22). As a control for a non-telomeric locus, we engineered an identical *URA3*-21xTus/*Ter* barrier into the *HIS2* locus on ChrVI (Figure 1A). Replication of a significant fraction of the right arm of ChrVI, including *HIS2* and *TEL06R*, generally originates from *ARS607* (23,24), and therefore the *his2* and *TEL06R* Tus/*Ter* barriers will usually be encountered by replication forks that derive from this origin. However, the timing of Tus-induced replication fork arrest would be expected to occur much later in S-phase in the strain with the *TEL06R* barrier. To ensure that induction of Tus leads to replication fork stalling at *TEL06R*, we performed 2DGE. This technique permits the detection of different types of DNA replication intermediates arising within a defined restriction fragment (25). We observed robust replication fork stalling in cells expressing Tus, but not in those lacking Tus (Figure 1B). These data indicate that the *Ter* sites alone do not lead to any detectable replication fork stalling, and that any replication perturbation arising due to fork stalling within the natural telomeric repeat sequences is relatively mild in comparison with that seen at the Tus/*Ter* barrier.

Previously, we reported that the *his2*::*URA3*-14xTus/*Ter* cassette could trigger distinct types of localized mutations in *URA3* (which confer resistance to 5-fluoro-orotic acid; 5-FOA) and that the Tus/*Ter*-induced *URA3* mutation rate was elevated ∼7-fold when the *SGS1* gene was deleted (15). We therefore compared *URA3* mutation rates for the *his2* (non-telomeric region) and *TEL06R* Tus/*Ter* barriers in wild-type (WT) and *sgs1* mutants. Following induction of the *his2* 21xTus/*Ter* barrier, the *URA3* mutation rate was increased 2.3-fold in WT cells, and 19-fold in *sgs1* mutants, as compared to the isogenic empty vector control (Figure 1C, left). Induction of the *TEL06R* Tus/*Ter* barrier caused an apparently higher rate of mutagenesis in each case (Figure 1C, right). Nevertheless, at both locations, loss of Sgs1 led to a consistent (∼8-fold) increase in the rate of Tus/*Ter*-induced *URA3* mutagenesis over that observed in WT cells. It should be noted that neither WT nor *sgs1* mutant strains harbouring a *his2* or *TEL06R* Tus/*Ter* barrier exhibited any obvious growth defects when Tus protein expression was induced.

To verify that the *TEL06R* phenotypes were due to replication fork stalling at Tus/*Ter*, we compared the effects of reversing the orientation of the 21x*Ter* module such that the Tus/*Ter* barrier, which we demonstrated previously to act in a polar manner (15), was in the ‘permissive’ (non-arresting) configuration. In this scenario, there was no detectable increase in 5-FOA resistant colonies (Supplementary Figure S1A and B), confirming that the Tus-dependent *URA3* mutagenesis is due to replication fork stalling. We also confirmed that spontaneous mutation rates at the unrelated *CAN1* locus on chromosome V were similar in WT and *sgs1* strains, irrespective of the location and status (i.e. on/off) of the Tus/*Ter* barrier (Figure 1D). It appears, therefore, that stalled replication forks at *TEL06R* result in a higher mutation rate than at a non-telomeric location, suggesting that the telomeric locus and/or the late timing of DNA replication might potentiate Tus/*Ter*-induced mutagenesis. Furthermore, loss of Sgs1 generates Tus-induced mutagenic events at both telomeric and non-telomeric loci, consistent with Sgs1 having a genome-wide role in limiting genomic alterations following replication fork stalling.

### Esc2 counteracts mutagenic events at a telomeric stalled fork

**Figure 2.**
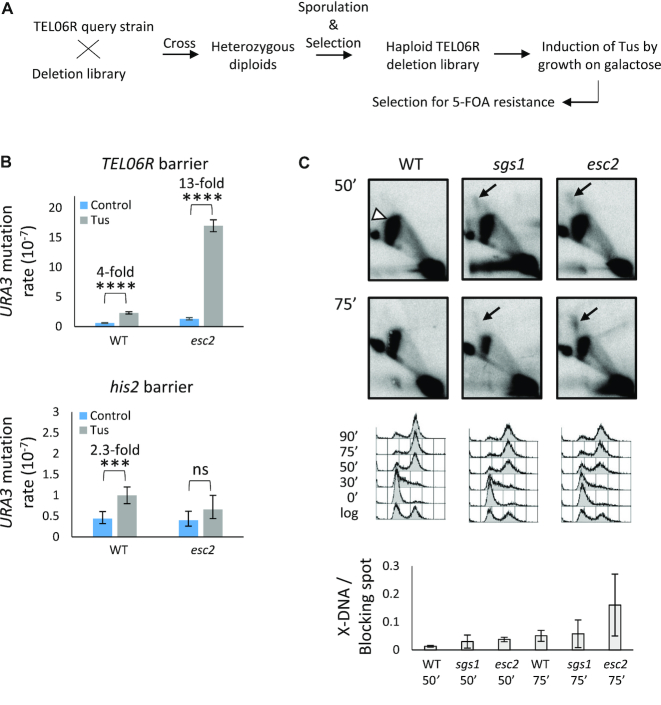
Esc2 suppresses *URA3* mutagenesis and X-DNA formation at the *TEL06R* Tus/*Ter* barrier. (**A**) Summary of the genome-wide screen set-up (see Supplementary Figure S1C and ‘Materials and Methods’ for details). (**B**) The effect of *ESC2* gene deletion on *URA3* mutagenesis at the *TEL06R* (top) and *his2* (bottom) Tus/*Ter* barriers. Data were analysed as described in Figure 1C.(**C**) WT, *sgs1* and *esc2* mutants harbouring the *TEL06R* Tus/*Ter* barrier were released from G1-arrest following induction of Tus. Genomic DNA was extracted at the indicated time points, and NheI-telomere fragments were analysed by 2DGE using a *TEL06R*-specific probe. The white arrowhead indicates replication fork stalling at the Tus/*Ter* barrier. Black arrows indicate unprocessed X-shaped DNA in *sgs1* and *esc2* mutants. Cell cycle profiles and quantification of the intensity of X-shaped DNA relative to that of the Tus-induced replication fork blocking spot are shown below. Quantifications were performed for three independent experiments, and error bars represent the standard deviation.

To identify factors that are important for responding to DNA replication stress in a telomeric region, we performed a high-throughput genetic screen to identify deletion mutants that exhibit an increase in *URA3* mutagenesis at the *TEL06R* Tus/*Ter* barrier (see ‘Materials and Methods’ section; Figure 2A and Supplementary Figure S1C). Positive hits from the screen (which we evaluated as potentially relevant to replication stress responses or telomeres) were then tested in the isogenic strain background harbouring a ‘permissive’ Tus/*Ter* cassette to define whether the observed *URA3* mutagenesis was dependent on replication fork stalling at Tus/*Ter*, or simply due to increased spontaneous *URA3* mutagenesis. We identified six initial hits from the screen that fulfilled these criteria (*sgs1, esc2, rad5, ioc3, rsc1* and *nup84*; an example of a typical plate from the screen is shown in Supplementary Figure S1D). Of note, Esc2 is an evolutionarily conserved ‘RENi’ family protein (26) that is implicated in the DNA replication stress response, HR repair, and in regulating telomeric chromatin architecture (27–31). For this reason, we primarily focused our efforts on characterizing the role of Esc2 at a stalled fork within a telomeric locus.

We first confirmed that expression of Tus did not alter the *CAN1* mutation rate in the *esc2* mutant (Supplementary Figure S1E). We then confirmed that loss of Esc2 caused an increased frequency of 5-FOA resistant colonies when the *TEL06R* Tus/*Ter* barrier was induced (Figure 2B, upper panel; Supplementary Figure S1F), and that this was dependent on the *Ter* sites being in the ‘restrictive’ configuration (Supplementary Figure S1G). Interestingly, however, this phenotype was not observed at the *his2* Tus/*Ter* barrier (Figure 2B, lower panel; Supplementary Figure S1F), suggesting that loss of Esc2 enhances mutagenesis selectively at a Tus/*Ter*-stalled fork in a telomeric region. Short telomeres and collapsed replication forks can be targeted to nuclear pore complexes (NPCs) for repair (32). Since the NPC component, *NUP84*, was also a hit in our screen, we investigated whether the specificity of Esc2 for *TEL06R* might be explained by alterations in the repair of stalled forks at NPCs. Because such repair involves the SUMO-targeted ubiquitin ligase, Slx5/8 (33,34), we also analysed a *slx8* mutant. However, upon re-testing the *nup84* and *slx8* mutants, we observed that the Tus-induced mutation frequency was only modestly enhanced and that these mutants behaved similarly to WT cells (Supplementary Figure S2A). Hence, *nup84* was a false-positive hit in our screen, and the study of these mutants was not pursued further.

To determine if this *esc2* mutant phenotype is dependent upon disruption of the native *TEL06R* X-element, we engineered strains with the *URA3*-21x*Ter* cassette integrated adjacent to, rather than within, the *TEL06R* X-element. In this strain, we also inactivated the *ARS610* replication origin located within the *TEL06R* X-element (henceforth referred to as the *ARS610Δ* Tus/*Ter* barrier) to ensure that forks originating from this origin could not rescue any *ARS607*-derived stalled forks at the Tus/*Ter* barrier (Supplementary Figure S2B). Similar to the *TEL06R* Tus/*Ter* barrier with a disrupted X-element, we observed that induction of the *ARS610Δ* Tus/*Ter* barrier caused elevated *URA3* mutagenesis when *ESC2* was deleted (or *SGS1* as a control; Supplementary Figure S2B). Therefore, Esc2 counteracts mutagenesis when a replication fork stalls within a region of *TEL06R* that is not confined solely to the very terminal regions of the chromosome.

To test if the observed phenotype was specific for *TEL06R*, we also compared the effects of engineering a *URA3*-21xTus/*Ter* barrier into a second telomere; *TEL07L* (Supplementary Figure S2C). In this case, we observed a higher background (i.e. Tus-independent) rate of resistance to 5-FOA in WT strains (Supplementary Figure S2C, lower panel). This background mutagenesis was less pronounced in the *esc2* mutant, likely reflecting telomere-specific differences in the ‘telomere position effect’ whereby telomeres can induce silencing of an adjacent gene (31,35). Nevertheless, induction of the *TEL07L* Tus/*Ter* barrier further increased the frequency of *URA3*, but not *CAN1* mutagenesis in an *esc2* mutant (Supplementary Figure S2C, lower panel). This suggests that Esc2 also normally counteracts replication-associated mutagenesis at the *TEL07L* Tus/*Ter* barrier. Taken together, we propose that Esc2 plays a general role in preventing mutations arising at a stalled replication fork in telomeric regions.

### Esc2 prevents abnormal DNA structures arising at the *TEL06R* Tus/*Ter* barrier

Using 2DGE, we demonstrated previously that Tus/*Ter*-induced replication fork stalling leads to the generation of abnormal ‘X-shaped’ DNA structures in *sgs1* mutants, which are indicative of unprocessed HR intermediates (13,14). To visualize replication intermediates arising at the *TEL06R* Tus/*Ter* barrier, WT, *sgs1* and *esc2* strains were released from G1 arrest, and the terminal *TEL06R* restriction fragment was analysed in each strain by 2DGE at 50 and 75 min after G1 release (Figure 2C). We observed that pronounced replication fork stalling was detectable at the *TEL06R* Tus/*Ter* barrier in all strains at both time points, despite the fact that bulk genome duplication was largely completed at these time points (Figure 2C, top and middle panels). This observation is consistent with the *TEL06R* locus being one of the last regions of the genome to be replicated (24). As expected, we observed that *sgs1* mutants accumulated X-DNA at the *TEL06R* Tus/*Ter* barrier (Figure 2C). Interestingly, X-DNA was also detectable in the *esc2* mutant, which became more pronounced in intensity at the later time point (75 min) (Figure 2C).

### Esc2 counteracts aberrant HR

**Figure 3.**
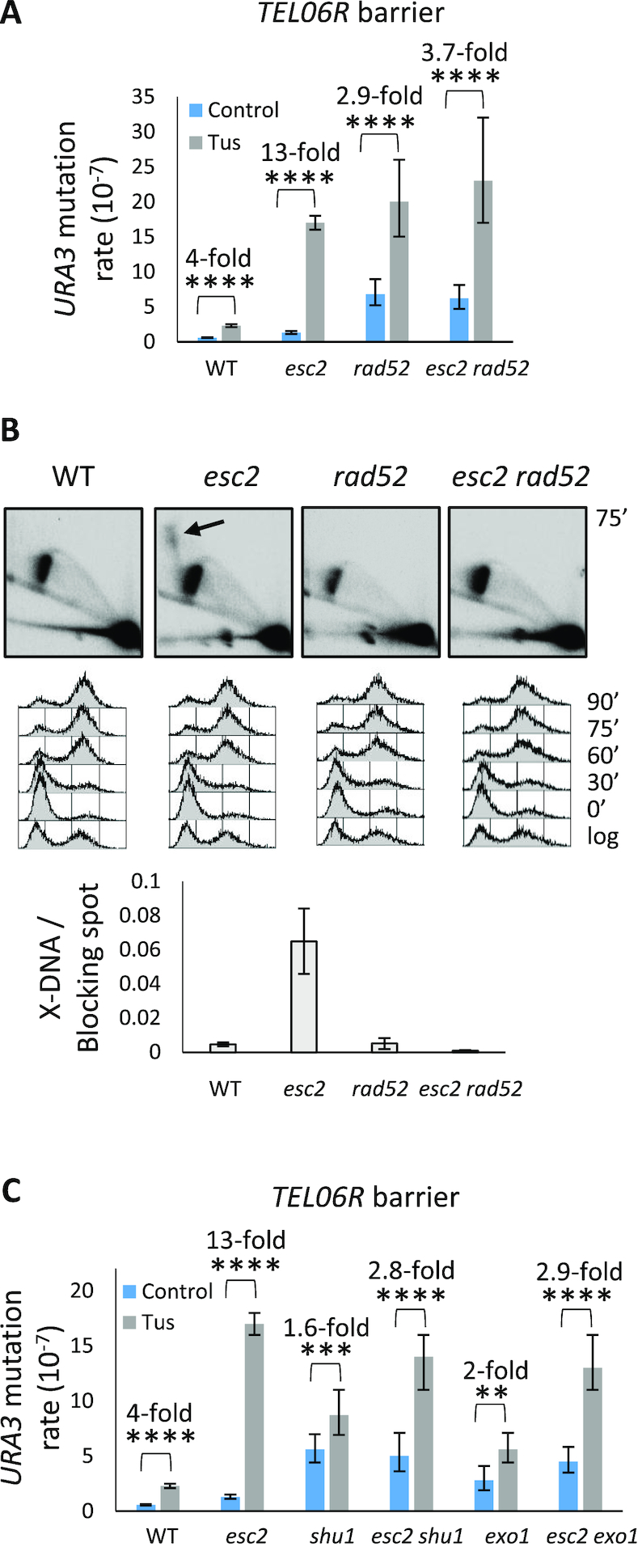
The mutagenesis and X-DNA accumulation in *esc2* mutants is dependent on HR. (**A**) The effect of deletion of *RAD52* on *URA3* mutagenesis at the *TEL06R* Tus/*Ter* barrier in an *esc2* mutant. *URA3* mutation rates were measured for the indicated strains harbouring the *TEL06R* Tus/*Ter* barrier. Data were analysed as described in Figure 1C. (**B**) Deletion of *RAD52* suppresses accumulation of X-shaped DNA in *esc2* mutants. Genomic DNA was extracted at 75′ after G1 release, and BspHI-telomere fragments were analysed by 2DGE and Southern blotting using *URA3-* and *TEL06R*-specific probes (two probes together). The black arrow indicates unprocessed X-DNA in the *esc2* mutant. Cell cycle profiles and quantification of the intensity of X-shaped DNA relative to that of the Tus-induced replication fork blocking spot are shown below. Quantifications were performed for three independent experiments, and error bars represent the standard deviation. (**C**) The effect of deletion of *SHU1* or *EXO1* on *URA3* mutagenesis at the *TEL06R* Tus/*Ter* barrier in an *esc2* mutant. *URA3* mutation rates were measured for the indicated strains harbouring the *TEL06R* Tus/*Ter* barrier. Data were analysed as described in Figure 1C.

Because Esc2 normally suppresses the accumulation of unresolved X-DNA at damaged replication forks (27,30), we investigated the role of HR factors in the Tus-induced mutagenesis at *TEL06R*. In agreement with previous results (36), we observed that deletion of *RAD51* or *RAD52* caused an increase in spontaneous mutagenesis (at both *URA3* and *CAN1*), and that induction of the Tus/*Ter* barrier caused a modest additional increase in *URA3* mutagenesis (Figure 3A and Supplementary Figure S3A–C). However, when the Tus/*Ter* barrier was induced in either *rad51 esc2* or *rad52 esc2* double mutants, there was no significant increase in the overall frequency of 5-FOA resistant colonies as compared to that observed in the *rad51* or *rad52* single mutants (Figure 3A and Supplementary Figure S3A–C and E). There was also no significant increase in replication fork stalling-associated (Tus-induced) *URA3* mutagenesis in the *esc2 rad52* double mutant, as compared to the respective *esc2* and *rad52* single mutants. This indicates an epistatic relationship between *esc2* and *rad52* in this assay (Figure 3A and Supplementary Figure S3D). As a consequence, the Tus-induced fold increase in *URA3* mutation rate in *esc2* cells was suppressed significantly by deletion of *RAD52* (Supplementary Figure S3E). Furthermore, deletion of *RAD52* in the *esc2* background also suppressed the accumulation of X-DNA at the *TEL06R* Tus/*Ter* barrier that is normally observed in an *esc2* single mutant (Figure 3B). Therefore, we propose that loss of HR suppresses the elevated *TEL06R* Tus/*Ter*-induced mutagenesis, and accumulation of aberrant HR intermediates, in *esc2* mutants. This could indicate that Esc2 acts in or regulates an HR pathway employed at stalled replication forks within telomeric DNA to limit the accumulation of recombination intermediates. Because Rad59-dependent break-induced replication is a mechanism by which yeast can perform recombinational telomere elongation (37), we also investigated the effects of deleting *RAD59*. However, deletion of *RAD59* did not suppress the elevated Tus/*Ter* mutagenesis in *esc2* mutants (Supplementary Figure S3A, bottom panel).

Our previous findings indicated that Tus/*Ter*-induced mutations at the *his2* 14xTus/*Ter* barrier are dependent on Shu1 and Exo1 (15). We therefore examined if this was also the case for the *TEL06R* 21xTus/*Ter* barrier. We observed that deletion of *SHU1* or *EXO1* produced the expected spontaneous mutator phenotypes (Figure 3C and Supplementary Figure S3F) (36,38), as well as a small increase in Tus/*Ter*-induced mutagenesis (Figure 3C). However, this phenotype was less pronounced than that observed in WT cells when assessing fold increase in mutation rates (Figure 3C and Supplementary Figure S3G). We also observed that deletion of *EXO1* or *SHU1* significantly reduced the rate of Tus-induced *URA3* mutagenesis in the *esc2* mutant (from 13-fold to <3-fold) (Figure 3C and Supplementary Figure S3G and H). Taken together, we propose that Esc2 counteracts mutations that arise due to aberrant HR following replication fork perturbation in a telomeric region and that these mutagenic events are at least partially dependent on Shu1 and Exo1.

### Esc2 limits truncations arising in a telomeric region following replication fork stalling

**Figure 4.**
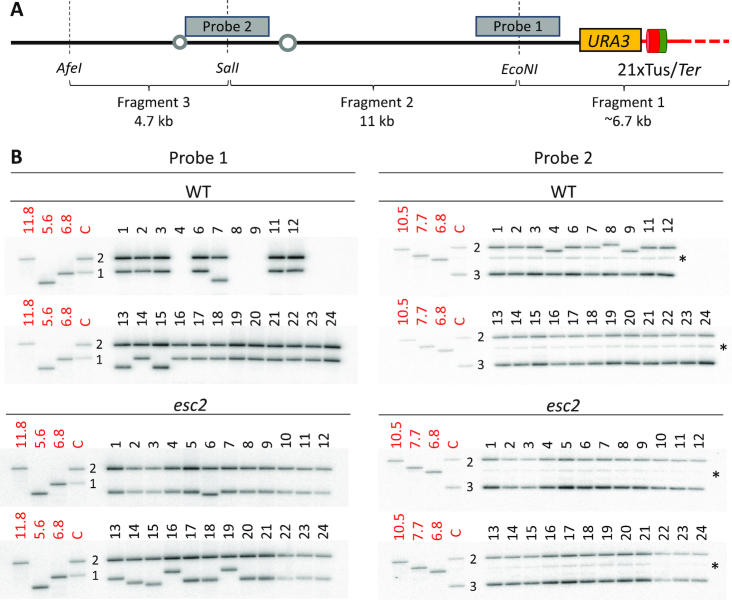
Replication fork stalling at the *TEL06R* Tus/*Ter* barrier triggers heterogeneous types of mutations. (**A**) Schematic illustrating *TEL06R* restriction fragments analysed by 1DGE (not drawn to scale). Dashed vertical lines indicate restriction enzyme sites. Probe 1 detects fragments 1 and 2. Probe 2 detects fragments 2 and 3. The positions of *URA3* and the Tus/*Ter* barrier as well as the size of the restriction fragments are indicated. (**B**) DNA was extracted from individual 5-FOA resistant clones and AfeI*-*SalI-EcoNI*TEL06R* fragments were analysed by 1DGE. Restriction fragments were visualized using either probe 1 (left panel) or probe 2 (right panel). WT clones are shown at the top and *esc2* clones at the bottom. Markers (in kilobases in red text) are indicated on the leftmost portion of each gel. C represents non-mutated, WT restricted DNA (yielding ‘Fragment 2’ (11 kb) and ‘Fragment 1’ (6.7 kb + telomeric repeats) for probe 1. The corresponding fragments for probe 2 are ‘Fragment 2’ (11 kb) and ‘Fragment 3’ (4.7 kb). The asterisk denotes a non-specific band detected in all samples with probe 2. Note that with probe 1, the lower of the two bands (Fragment 1) in most *esc2* clones is smaller than that in WT clones.

**Figure 5.**
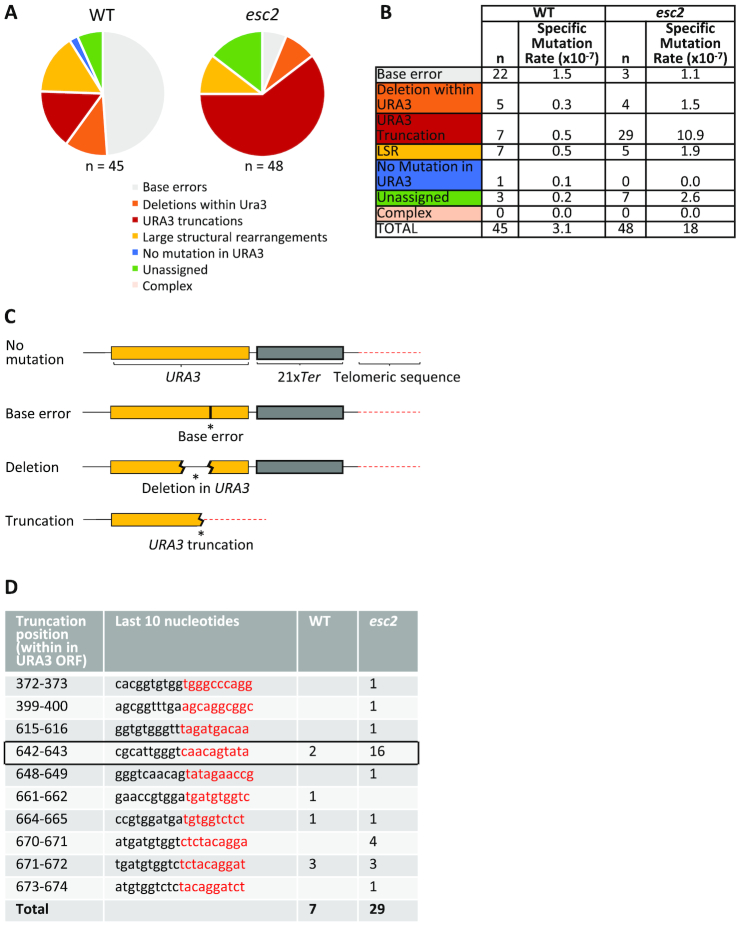
*esc2* mutants accumulate ‘truncations’ at the *TEL06R* Tus/*Ter* barrier. (**A**) Mutation types were identified at the *TEL06R* barrier in WT (left) and *esc2* (right) strains. Pie charts indicate the relative proportions of mutation types, as indicated in the key below. Data were obtained by restriction digest analysis and telomere tailing/sequencing. (**B**) The specific mutation rate for individual types of mutations was calculated. *n* = number of times a specific mutation type was observed. Colour coding corresponds to pie chart in (A). (**C**) Schematic illustration of the mutation types (base errors, deletions within *URA3* and *URA3* truncations) identified by sequencing. (**D**) A specific type of chromosomal truncation is prevalent in *esc2* mutants upon replication fork stalling at a telomere. Table showing the position (within the *URA3* ORF) from where DNA sequence is lost and the number of clones displaying the given truncation. Data were obtained by telomere tailing and DNA sequencing. Black sequence is retained, whereas red sequence is lost. Note that 16 of 29 *esc2* clones show an identical breakpoint (indicated by a black box).

To analyse the types of *ura3* mutations induced by replication fork arrest at *TEL06R* Tus/*Ter*, we extracted genomic DNA from 45 WT and 48 *esc2* 5-FOA resistant clones. For each of the clones, we performed Southern blot analysis to probe the integrity of the chromosome VI arm (Figure 4A and B; Supplementary Figure S4A). In WT cells, we observed that 7 of the 45 clones exhibited apparent loss of the terminal 6.7 kb EcoNI fragment detected by probe 1, while fragment 2 in most cases was reduced in size and only detected by probe 2 (Figure 4B and Supplementary Figure S4A). This suggests that complex genome rearrangements can occur outside of the *URA3*-21x*Ter* module (henceforth defined as ‘large structural rearrangements’ or LSRs) following fork stalling in WT cells. The remainder of the WT clones (38 of 45) exhibited either a smaller deletion in fragment 1, or else no detectable change in restriction fragment size when using this assay. Using a modified ‘telomere-tailing’ assay to amplify the terminal ∼2.5 kb *TEL06R* region, we were able to detect mutations by DNA sequencing in the majority of those remaining 38 clones. In cases where the ‘telomere-tailing’ and sequencing procedure failed to give a clear result, the mutation was designated as ‘unassigned’ (3 clones) (Figure 5A and B). The sequencing results allowed us to unequivocally assign mutation types as either base errors (22 clones), 22-147-bp deletions in *URA3* (with retention of the *Ter* sites; 5 clones), or truncations (7 clones) (Figure 5A–C). The latter class appear as ∼1.2 kb deletions in fragment 1 using the restriction digest mapping assay (Figure 4B and Supplementary Figure S4A) and comprise at least four different events in which the *Ter* array was lost and new telomeric sequence appears within the *URA3* ORF (Figure 5C and D).

Even though the proportion of mutations assigned as base errors was much lower in *esc2* than in WT cells, the specific base error mutagenesis rates were very similar (Figure 5B). However, in contrast to what was observed in WT cells, truncations comprised the vast majority of mutation types in the *esc2* mutant (29 of the 48 clones analysed) (Figure 4B; Supplementary Figure S4A; Figure 5A and B). Indeed, the rate of truncation mutagenesis was 21-fold higher in the *esc2* mutant than in the WT cells (10.9 × 10^−7^ in *esc2*, as compared to 0.5 × 10^−7^ in WT; Figure 5B), suggesting that Esc2 normally prevents this specific type of mutation from arising at the *TEL06R* Tus/*Ter* barrier. The remainder of the mutation types in *esc2* mutants were either large structural rearrangements (5 clones), base errors (3 clones) or 116-1125-bp deletions in *URA3* (that retained the *Ter* sites; 4 clones) (Figure 5A and B). DNA sequencing of 29 truncation events in *esc2* mutants revealed 9 distinct truncation positions (Figure 5D). Nevertheless, 16 of these truncations exhibited an identical position (located 301 bp from the first *Ter* site), which contains a putative telomere seed sequence, suggesting a strong bias for chromosomal truncations at this site in *esc2* mutants accompanied by *de novo* telomere addition (Figure 5D and Supplementary Figure S4B). Taken together, we propose that DNA replication stress in a late-replicating telomeric region can generate various types of genome rearrangements, including large structural rearrangements, deletions and chromosomal truncations. Furthermore, chromosomal truncations are the predominant class of Tus/*Ter*-induced mutations in *esc2* mutants, suggesting that Esc2 strongly counteracts the mutagenic processes that lead to telomere-proximal truncations.

### Loss of Mph1 or Rad52 suppresses aberrant HR at a telomeric stalled fork in *esc2* mutants

**Figure 6.**
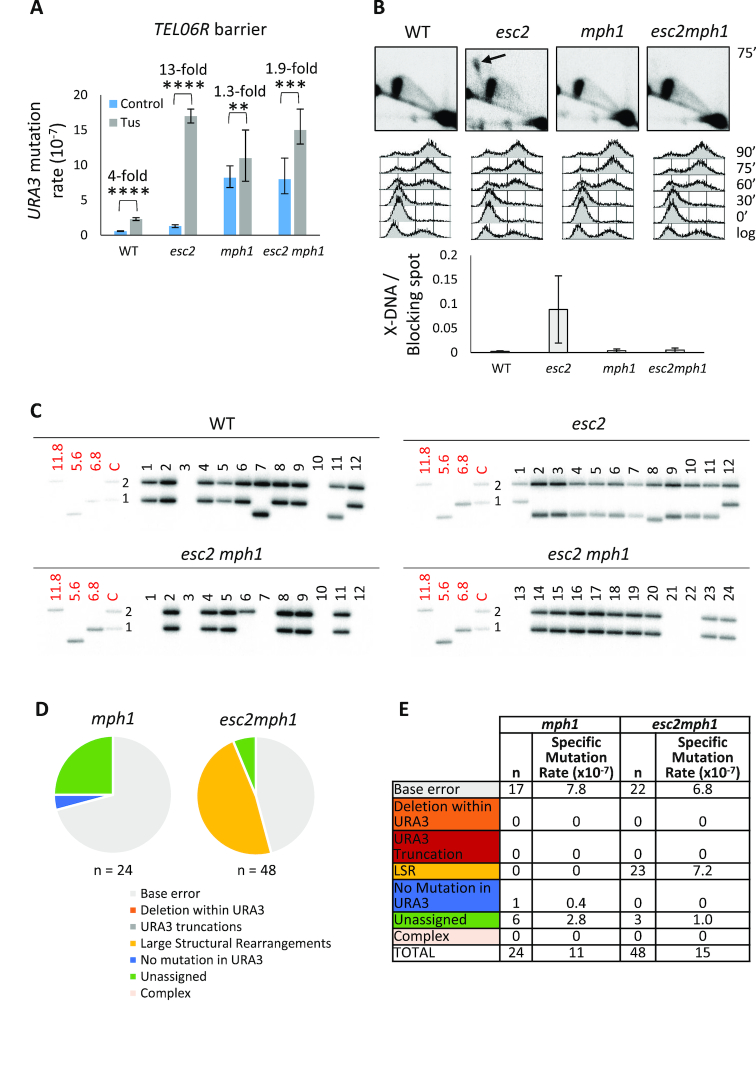
Abnormalities in *esc2* strains are suppressed by deletion of *MPH1*. (**A**) Deletion of *MPH1* suppresses *URA3* mutagenesis at the *TEL06R* Tus/*Ter* barrier. Data were analysed as described in Figure 1C. (**B**) Deletion of *MPH1* suppresses accumulation of X-shaped DNA in *esc2* mutants. Genomic DNA was extracted at 75′ after G1 release, and BspHI-telomere fragments were analysed by 2DGE and Southern blotting using *URA3-* and *TEL06R*-specific probes (two probes together). The black arrow indicates unprocessed X-DNA in the *esc2* mutant. Cell cycle profiles and quantification of the intensity of X-shaped DNA relative to that of the blocking spot are shown below. Quantifications were performed for three independent experiments, and error bars represent the standard deviation. (**C**) DNA was extracted from individual 5-FOA resistant clones and SalI-EcoNI*TEL06R* fragments were analysed by 1DGE using probe 1 shown in Figure 4A. WT and *esc2* clones are shown in the upper panels, while *esc2 mph1* clones are shown in the lower panels. Markers (in kilobases) are as shown in Figure 4B. (**D**) Mutation types were identified at the *TEL06R* barrier in the *mph1* (right) and *esc2 mph1* (left) mutant. The pie charts indicate the relative proportions of mutation types, as indicated in the key below. Data were obtained by restriction digest analysis and telomere tailing/sequencing. (**E**) The specific mutation rate for individual types of mutations was calculated. *n* = number of times a specific mutation type was observed. Colour coding corresponds to pie chart in (D).

Previous reports have suggested a role for Esc2 in regulating the activity of the Mph1 helicase at sites of DNA damage (27,39). We therefore investigated whether deletion of *MPH1* might influence telomere instability resulting from replication fork arrest in *esc2* mutants. Consistent with previous studies, we observed that the background frequency of mutations was elevated in a *mph1* mutant (Figure 6A and Supplementary Figure S4C) (40). However, the Tus-induced mutagenesis normally observed in *esc2* cells was significantly suppressed by *MPH1* deletion (Figure 6A and Supplementary Figure S4D and E). Moreover, the persistence of X-shaped DNA at the Tus/*Ter* barrier in *esc2* cells was strongly reduced when Mph1 was absent (Figure 6B), as was the high rate of truncations characteristic of *esc2* mutants (Figure 6C–E and Supplementary Figure S4F). In *mph1* cells, base errors were the most frequent type of mutation, and the base error rate was highly similar for *mph1* and *mph1 esc2* strains (Figure 6D and E). The frequency of large structural rearrangements was higher in the *esc2 mph1* double mutant than in WT or either of the *esc2* or *mph1* single mutants, but all of the rearrangements resembled those seen in WT cells with loss of fragment 1 (Figure 6C and Supplementary Figure S4F).

We also investigated how the mutation spectrum in an *esc2* mutant was influenced by deletion of *RAD52*. As with deletion of *MPH1*, we observed that the characteristic high level of *URA3* truncation events in *esc2* cells was suppressed by deletion of *RAD52*. Indeed, the mutation spectra for the *rad52* and *esc2 rad52* mutants were highly similar, with the majority of mutations being base changes in *URA3* (Supplementary Figure S5A). In the *rad52* and *esc2 rad52* mutants, even though not very frequent, we observed a type of mutation that was not detected in WT or *esc2* cells. This mutation type consisted of more than one base error within an eight base-pair region and was termed ‘complex’ (Supplementary Figure S5A). Taken together, these data indicate that elevated chromosomal truncations arising in *esc2* mutants are due to the aberrant activity of Mph1 and the HR machinery at a telomeric stalled replication fork.

### Srs2 influences HR pathways at stalled telomeric replication forks

Esc2 was shown previously to facilitate recombination-mediated DNA damage tolerance by allowing optimal recruitment of Rad51 to sites of damage by promoting turnover of the ‘anti-recombinase’, Srs2 (29). To assess whether the phenotypes observed at the *TEL06R* barrier were regulated by Srs2, we determined the *URA3* mutation rate at the *TEL06R* and *his2* Tus/*Ter* barriers in *srs2* mutant cells. Fluctuation tests showed that loss of *SRS2* resulted in an increase in *URA3* mutation rate at *TEL06R* compared to that seen in WT cells, an effect that was absent at the *his2* barrier (Supplementary Figure S5B). However, when we investigated the mutation spectrum, we found that the predominant mutation types in *srs2* cells at the *TEL06R* Tus/*Ter* barrier were base errors and deletions within *URA3* (Supplementary Figure S5C). In contrast to what was seen in WT or *esc2* mutants, we did not detect any *URA3* truncations in the *srs2* mutant (Supplementary Figure S5C). Taken together, our data might suggest that Srs2 activity is involved in determining which HR sub-pathway is deployed at a telomeric stalled fork. In the absence of Srs2, HR is known to be dysregulated, but in our system this does not lead to an elevated frequency of truncations at the *TEL06R* Tus/*Ter* barrier. Instead, loss of Srs2 selectively leads to an increase in deletions arising behind the telomeric stalled fork. Due to a synthetic growth defect of the *esc2 srs2* double mutant, it was not possible to examine the phenotype of this double mutant in our study.

## DISCUSSION

In this study, we analysed the consequences of DNA replication fork stalling in a telomeric region (*TEL06R*) through the use of the genetically tractable Tus/*Ter* barrier (13,14). We have demonstrated that this telomeric Tus/*Ter* barrier can induce pronounced mutagenesis, which is associated with at least three distinct types of chromosomal rearrangements: (i) large structural rearrangements (which can extend to over many kilobases in some cases), (ii) localized deletions in the ∼1 kb region behind the stalled replication fork and (iii) chromosomal truncations followed by telomeric sequence. Two features of a telomere may predispose to these types of genome rearrangements following replication fork arrest. First, the telomeric Tus/*Ter* construct was engineered to ensure that no downstream origin could be activated to rescue the stalled replication fork. Second, the *TEL06R* locus is one of the very last regions of the genome to be replicated (24). Therefore, any unresolved DNA replication/HR intermediates may be carried into mitosis, and be subjected to the physical forces of the mitotic spindle (41). Indeed, this latter consideration might explain how at least some of the large structural rearrangements arise. To counteract these challenges, we propose that cells utilize specific mechanisms to regulate telomere DNA replication and recombination, thus ensuring optimal telomere stability.

We performed a genome-wide screen to identify factors that protect against replication-induced mutagenesis, and identified a number of proteins that counteract mutagenesis when DNA replication stress occurs in a telomeric region. In particular, we identified Esc2 as an ‘anti-mutagenic’ factor in the screen, and confirmed that loss of this protein significantly enhances mutagenesis at the *TEL06R* Tus/*Ter* barrier. Esc2 is an evolutionarily conserved SUMO-like domain protein (26), which functions in the DNA replication stress response, HR repair, and establishment of telomeric chromatin architecture (27–31). Interestingly, however, the anti-mutagenic effects of Esc2 are only evident at the telomeric Tus/*Ter* barrier, suggesting that the combined loss of Esc2’s ability to regulate repair of damaged replication forks and maintain telomeric chromatin architecture lead to effects that are much more pronounced within telomeric DNA. Further characterization of this phenotype revealed that chromosomal truncations are the most prominent Tus/*Ter*-induced mutation type occurring in *esc2* mutants, indicating that Esc2 has a specialized role in preventing these events at telomeric loci following DNA replication stress. It is possible that the role of Esc2 in suppressing truncations at non-telomeric loci may be equally important. However, since the types of truncations seen at *TEL06R* might be lethal at *his2* due to loss of essential genes, we would be unable to score this type of mutation in our assays. In this context, it would appear unlikely that mutants with elevated levels of Tus-induced mutagenesis at both *TEL06R* and *his2*, such as *sgs1*, would phenocopy the strong bias for chromosomal truncations observed in *esc2* mutants.

**Figure 7.**
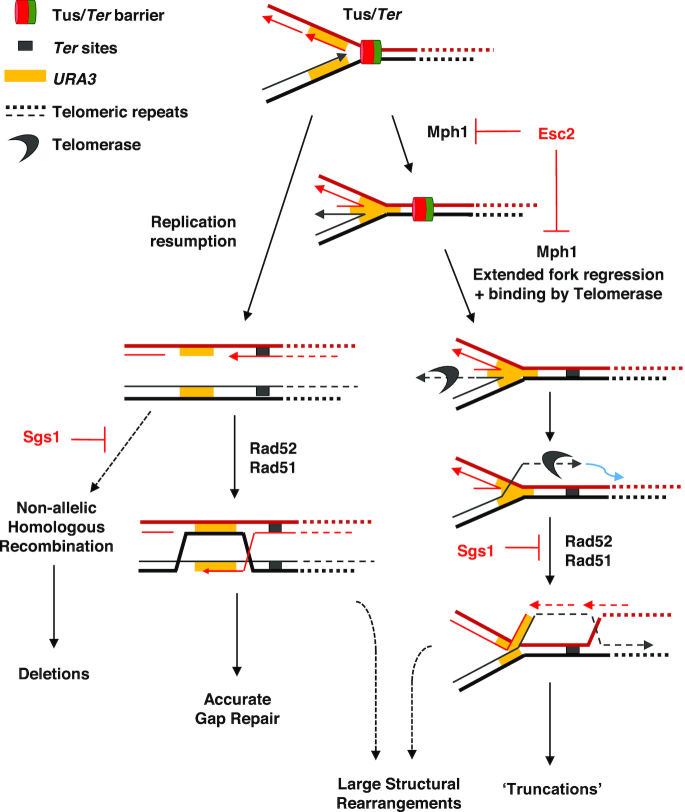
Model for repair of a telomeric stalled replication fork. A replication fork is stalled at the Tus/*Ter* barrier (top). Resection of the lagging strand followed by DNA replication resumption would leave a ssDNA gap behind the fork (depicted on the left). The gap can be filled by post-replicative HR mediated by the Rad51 and Rad52 proteins amongst others. Deletions may arise via non-allelic HR repair of the ssDNA gap, in a process that is counteracted by Sgs1’s ability to disrupt aberrant strand invasion events. Alternatively, extended or prolonged fork regression might occur that is mediated by Mph1 (depicted on the right). Binding of Telomerase and the addition of telomeric repeats to the regressed fork could create a recombinogenic substrate that engages in HR with the telomeric repeats downstream of the *Ter* sites. Resolution of this HR intermediate would result in the observed telomere-proximal truncations observed in *esc2* mutants. Large structural rearrangements could arise if unresolved HR intermediates are carried into mitosis and undergo chromosome breakage. Loss of Esc2 is proposed to lead to dysregulation of Mph1 activity, resulting in more extensive fork regression. Srs2 might normally serve to prevent HR intermediates from being processed correctly, with the result that *srs2* cells funnel intermediates preferentially down the left branch of the pathway.

Given that Esc2 is a SUMO-like RENi protein that lacks any detectable enzymatic activity, it likely functions as an adaptor protein that regulates the activity of other key DNA-metabolizing enzymes either directly or through binding to branched DNA structures (29). Candidate proteins that cooperate with Esc2 directly at telomeric stalled forks include Srs2 and Mph1. Indeed, loss of Srs2 caused dysregulated HR at the *TEL06R* barrier, as well as an altered mutation spectrum compared to that observed in either WT or *esc2* mutants. This observation is consistent with the proposed role for Esc2 in regulating Srs2 anti-recombinase activity at stalled forks (29). However, it is clear that a loss of regulation of Srs2 *per se* cannot fully explain the range of phenotypes that we observe in an *esc2* mutant; most notably, because *esc2* and *srs2* mutants display very different Tus-induced mutation spectra. The other candidate examined in this study was Mph1, which processes distinct types of HR intermediates (e.g. regressed forks and D-loops) following DNA damage (27,31,42). Because Tus/*Ter*-induced mutagenesis at *TEL06R* in *esc2* cells is Mph1-dependent, and deletion of *MPH1* eliminates the associated accumulation of HR-derived X-DNA, we propose that chromosomal truncations at the *TEL06R* barrier most likely arise due to dysregulated Mph1 activity at this stalled replication fork. It was recently demonstrated that negative regulation of Mph1 is important for cells to avoid an accumulation of toxic HR intermediates generated by uncontrolled fork reversal/regression (43,44). This proposal is consistent with our observation that the elimination of HR and Mph1 suppresses chromosomal truncations and X-DNA in *esc2* mutants. Mph1 can also promote telomere uncapping and the accumulation of single-stranded DNA (ssDNA) at telomeres when overexpressed in the absence of Telomerase (45). Hence, to maintain stable telomeres, Mph1 must be tightly regulated. We therefore propose a model (Figure 7) that is consistent with the previous and current data whereby Mph1 promotes the regression of the replication fork stalled at the telomeric Tus/*Ter* barrier, and that Esc2 regulates this process. In WT cells, fork regression is restrained optimally. In contrast, we propose that unrestrained fork regression occurring in the absence of Esc2 allows telomerase to gain access to the exposed DNA end of the regressed fork, and hence add telomeric TG repeats (Figure 7, right pathway). This would subsequently create a substrate able to engage in HR with the native TG repeats downstream of the *Ter* sites, and thereby drive formation of the observed truncations. Consistent with this model is the finding that a strong hotspot for truncation mutations is seen in *esc2* mutants. The sequence context in which this hotspot is embedded is notable for being highly enriched in TG residues (Supplementary Figure S4B), which offers the potential for preferential association with Telomerase. However, the TG-rich nature of this hotspot might in itself be sufficient to directly promote HR with the telomeric DNA. An alternative model would be that the regressed fork is subjected to cleavage by a nuclease, followed by *de novo* telomere addition to generate truncations. Esc2 has been reported to interact with and stimulate the activity of the Mus81 endonuclease (46). Loss of Mus81 regulation could potentially promote uncontrolled cleavage of regressed replication forks. If this latter scenario were true, it would suggest the involvement of the HR machinery directly at the stalled fork, most likely through promoting fork regression. It would be interesting in the future to identify mutations that disrupt the interaction between Esc2 and Mus81, and determine how they influence the rate of Tus-induced truncation mutagenesis.

Several reported activities of Esc2 may be relevant to the telomere instability that we have observed in this study. First, Esc2 interacts with Sir2 (31), which is highly enriched at telomeric chromatin (47). Second, many proteins are SUMOylated in the context of replication stress (48) and Esc2 physically interacts with Ubc9 and SUMO (30), suggesting that Esc2 could respond to stalled replication by binding to SUMOylated proteins at the fork. In this context, it is interesting to note that Esc2 promotes an accumulation of SUMOylated substrates of the SUMO ligase (Mms21) within the Smc5/6 complex, and cooperates with Mms21 to suppress gross chromosomal rearrangements (28), possibly through a mechanism that involves the ability of the Smc5–Smc6 complex to block fork regression by Mph1 (28,44,49–50). Finally, short telomeres and collapsed replication forks can be targeted to the NPCs for repair (32). Even though the *nup84* and *slx8* mutants did not give a clear mutagenic phenotype in our strain background, it remains a possibility that the role of Esc2 at stalled forks in telomeric loci is conducted in the context of NPCs.

Our findings link DNA replication stress and HR repair, and therefore further analysis of the *TEL06R* Tus/*Ter* barrier could lead to a better understanding of how these biological processes cooperate to promote telomere stability. In future studies, it will be of interest to examine the potential telomeric roles of NIP45 (an Esc2 homologue) in human cell lines. The roles of NIP45 are poorly characterized and have focussed largely on putative roles in immune cell transcription (51,52). Future studies should investigate the consequences of impairing the function of NIP45 on telomere stability in both telomerase-positive and ALT-maintained cancer cell lines.

## Supplementary Material

Supplementary Data

## Notes

Present address: Nicolai B. Larsen, Center for Protein Research, University of Copenhagen, Panum Institute, 2200 Copenhagen N, Denmark.
