# Supplementary material for: Esc2 promotes telomere stability in response to DNA replication stress
Source: Nucleic Acids Res. 2019 Mar 6;47(9):4597–611. doi: 10.1093/nar/gkz158 (PMC6511870; doi:10.1093/nar/gkz158)
Supplement: Supplementary Data [file gkz158_supplemental_file.pdf]

Supplementary Figure 1

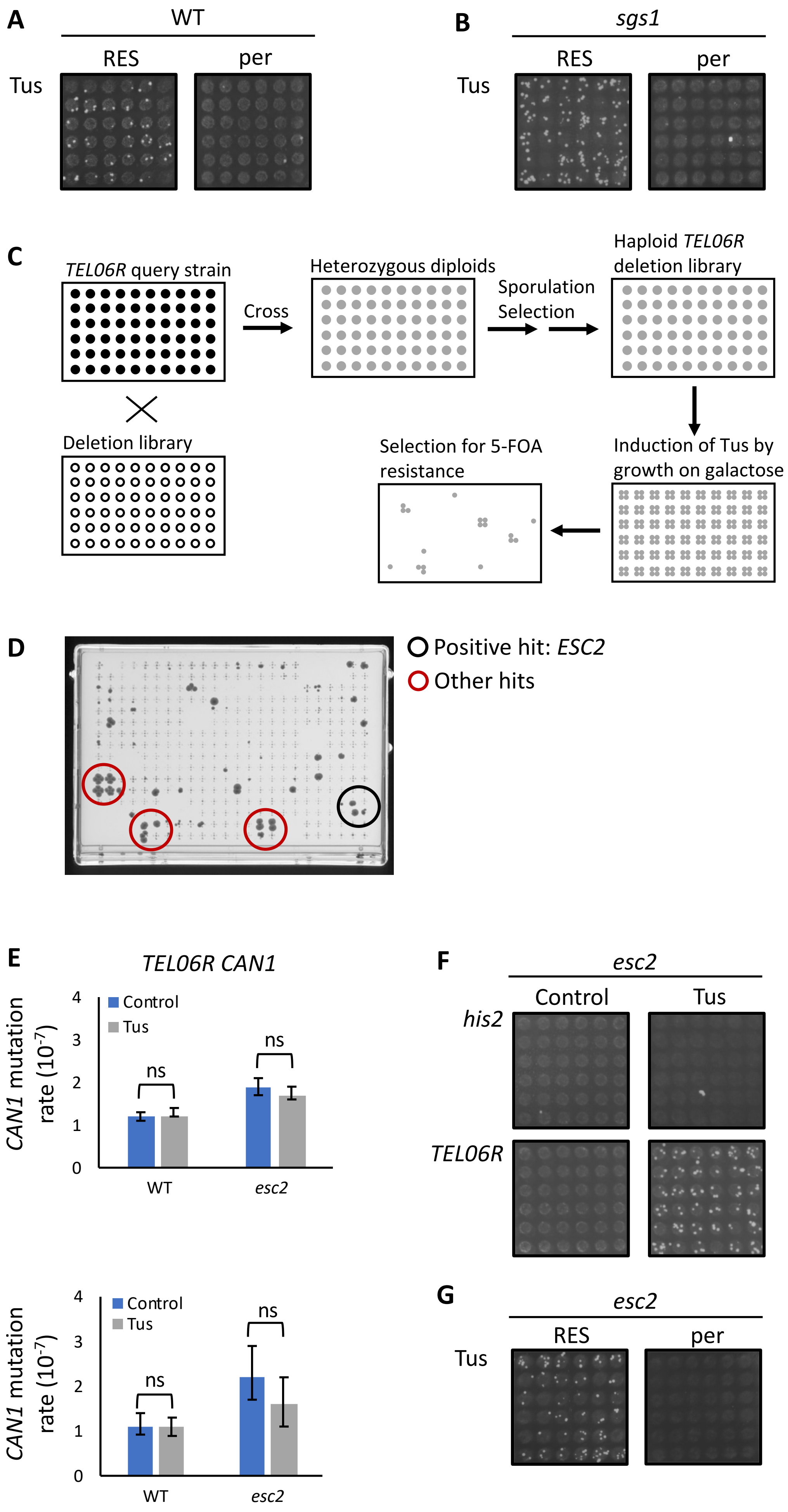

Supplementary Figure 1. Screening for factors that limit *TEL06R* Tus-induced *URA3* mutagenesis. **(A-B)** Effect of the orientation of *Ter* sites on *URA3* mutagenesis at the *TEL06R* Tus/*Ter* barrier in WT (**A**) and *sgs1* (**B**) cells. The *Ter* sites placed in either the restrictive (RES; left) or permissive (per; right) orientation relative to *ARS607.* Tus was expressed in both cases. Strains were grown on non-selective, galactose-containing plates to induce the Tus/*Ter* barrier, before selection for resistance to 5-FOA. **(C)** Genome-wide screen setup. A query strains harbouring the *TEL06R* Tus/*Ter* barrier was crossed with the *S. cerevisiae* gene deletion library. Heterozygous diploids were sporulated on plates, followed by multiple steps of selection for haploids harbouring both the *TEL06R* Tus/*Ter* barrier and the given gene deletion. Induction of Tus by transfer to galactose-containing media was followed by selection for 5-FOA resistance. For the last two steps, each gene deletion was spotted four times and hits were scored as those showing growth on at least 3 of the 4 spots. **(D)** Example of a 5-FOA result plate. The black circle indicates the positive hit; *esc2*. Red circles indicate hits that were either found likely to be unrelated to the Tus-induced replication fork stalling due to their function (and were not tested further) or showed no Tus-dependent effect when tested manually. **(E)** The effect of *ESC2* gene deletion on *CAN1* mutagenesis was tested in strains with either the *TEL06R* (top) or the *his2* (bottom) Tus/*Ter* barrier. Data were analysed as described in Figure 1B. **(F)** The effect of *ESC2* gene deletion on *URA3* mutagenesis at a Tus/*Ter* barrier located either at *his2* (upper) or *TEL06R* (lower). Indicated strains were grown on non-selective, galactose-containing plates to induce the Tus/*Ter* barrier, before selection for 5-FOA resistance. In both cases, examples without (left) and with (right) Tus expression are shown. **(G)** The effect of *ESC2* gene deletion on *URA3* mutagenesis at the *TEL06R* Tus/*Ter* barrier placed in either the restrictive (RES; left) or permissive (per; right) orientation relative to *ARS607.* Indicated strains were grown on non-selective, galactose-containing plates to induce the Tus/*Ter* barrier, before selection for 5-FOA resistance.

Supplementary Figure 2

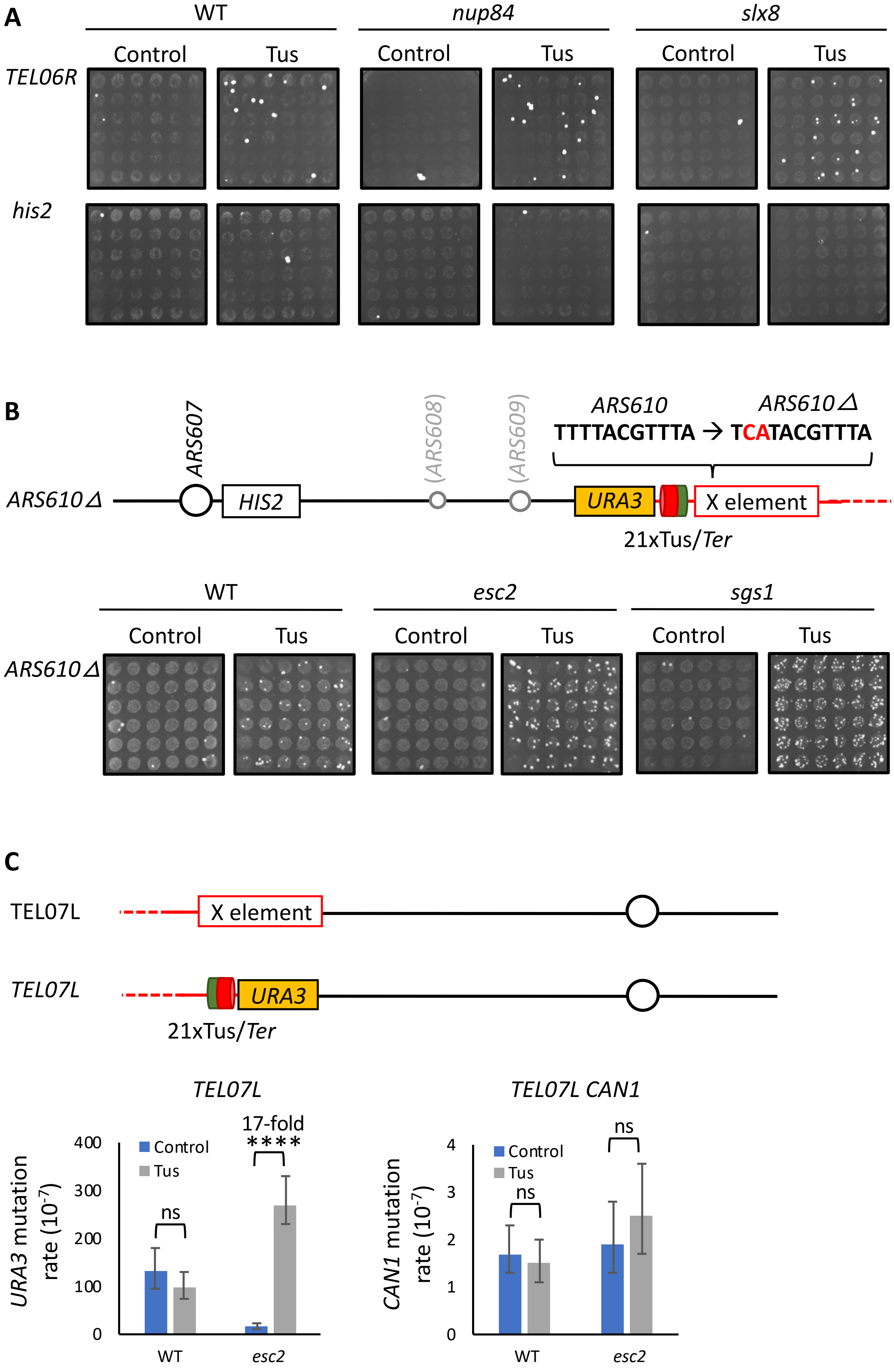

Supplementary Figure 2. Mutagenesis in *esc2* cells is not limited to the *TEL06R* Tus/*Ter* barrier where the X-element has been deleted. **(A)** The effect of *NUP84* and *SLX8* gene deletion on *URA3* mutagenesis at a Tus/*Ter* barrier located either at *TEL06R* (upper) or *his2* (lower). Indicated strains were grown on non-selective, galactose-containing plates to induce the Tus/*Ter* barrier, before selection for 5-FOA resistance. In both cases, examples without (left) and with (right) Tus expression are shown. **(B)** Schematic diagram showing the *ARS610Δ* Tus/*Ter* construct. *URA3*-21x*Ter* was inserted centromere-proximal to the X element of *TEL06R*, and *ARS610* was inactivated by a 2-bp change shown above (in red). In the lower section, *URA3* mutagenesis was tested in the indicated strains at the *ARS610Δ* Tus/*Ter* barrier. Indicated strains were grown on non-selective, galactose-containing plates to induce the Tus/*Ter* barrier, before selection for 5-FOA resistance. **(C)** Schematic diagram showing the *TEL07L* Tus/*Ter* construct. The X element was substituted for the *URA3-21x*Tus/*Ter* barrier. In the lower section, the effect of *ESC2* gene deletion on *URA3* (left) and *CAN1* (right) mutagenesis at the *TEL07L* Tus/*Ter* barrier was tested. Data were analysed as described in Figure 1C.

Supplementary Figure 3

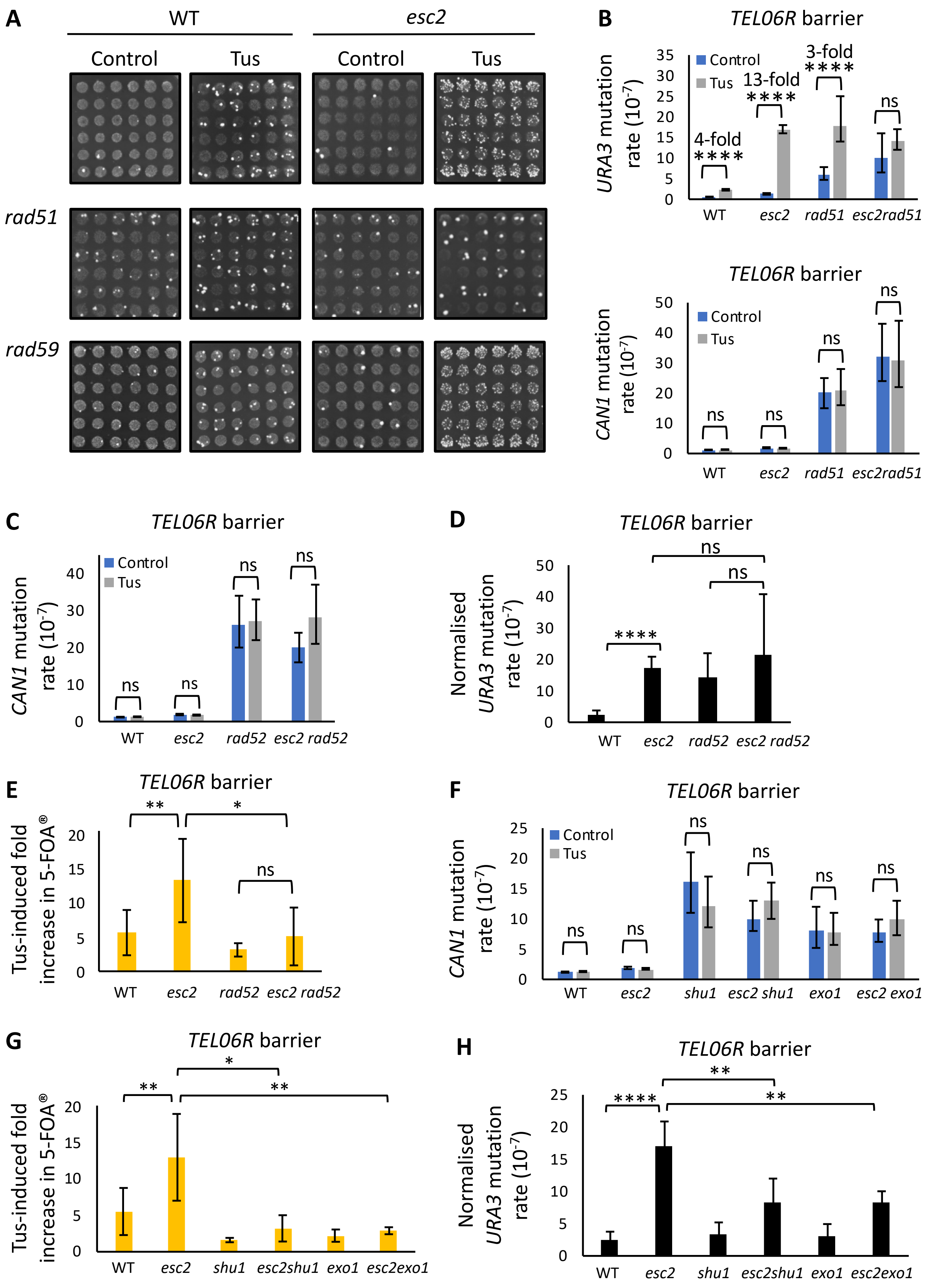

Supplementary Figure 3. The *esc2* mutagenesis phenotype is dependent on HR. **(A)** Effect of deletion of *RAD51* and *RAD59* on *URA3* mutagenesis. Indicated strains were grown on non-selective, galactose-containing plates to induce the Tus/*Ter* barrier, before selection for 5-FOA resistance. **(B)** Effect of *RAD51* gene deletion on *URA3* (top) and *CAN1* (bottom) mutagenesis at the *TEL06R* Tus/*Ter* barrier was tested. Data were analysed as described in Figure 1C. **(C)** Effect of *RAD52* gene deletion on *CAN1* mutagenesis at the *TEL06R* Tus/*Ter* barrier was tested. Data were analysed as described in Figure 1C. **(D)** The normalised *URA3* mutation rate at *TEL06R* in cells expressing Tus was calculated after subtraction of the background level (in the control lacking Tus). Error bars represent standard deviation from three independent experiments. Statistical analysis of differences was performed using an unpaired t-test and significance is indicated when $p<0.05$. **(E)** Tus-induced fold increase in *URA3* mutation rate (fold increase in 5-FOA resistance (5-FOA®)) was plotted for the indicated strains. Error bars represent standard deviation from three independent experiments. Statistical analysis of differences was performed using an unpaired t-test and significance is indicated when $p<0.05$. **(F)** Induction of the Tus/*Ter* barrier does not affect *CAN1* mutation rates in *shu1*, *esc2 shu1*, *exo1* and *esc2 exo1* strains. *CAN1* mutation rates were measured for the indicated strains harbouring the *TEL06R* Tus/*Ter* barrier. Data were analysed as described in Figure 1C. **(G)** Tus-induced fold increase in *URA3* mutation rate was plotted for the indicated strains. Data were analysed as in (E). **(H)** Normalised *URA3* mutation rate at *TEL06R* is plotted where background level (control) is subtracted from the Tus-induced mutation rate. Data were analysed as in (D).

Supplementary Figure 4

A

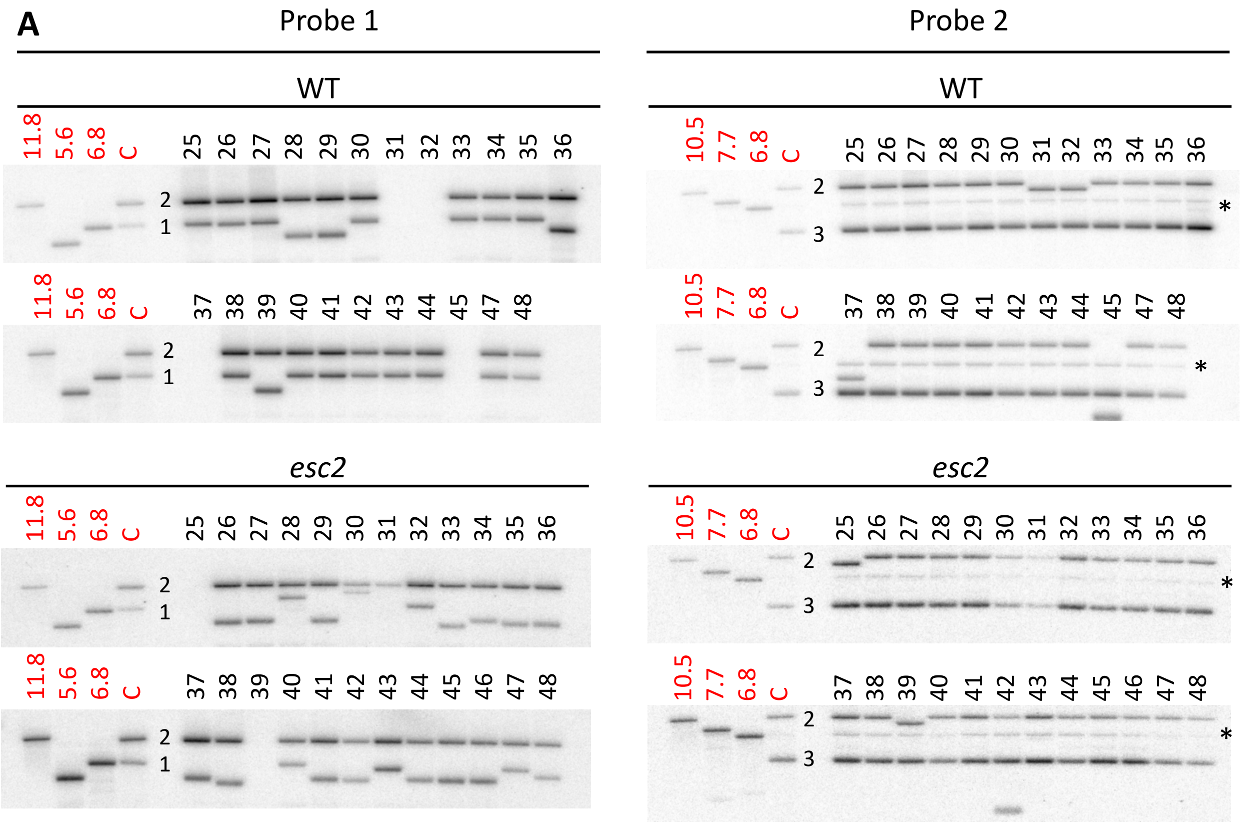

B

Common truncation breakpoint*

5'-ctttattgctcaaagagacatgggtggaagagatgaaggtt acgattggttgattatgacacccggtgtgggtttagatgacaag ggagacgcattgggt*caacagtatagaaccgtggatgatgtg gtctctacaggatctgacattattattgttggaagaggactattt gcaaagggaagggatgctaaggtagagg-3'

Position 642*643 in URA3 ORF
Gly214*Gln215

C

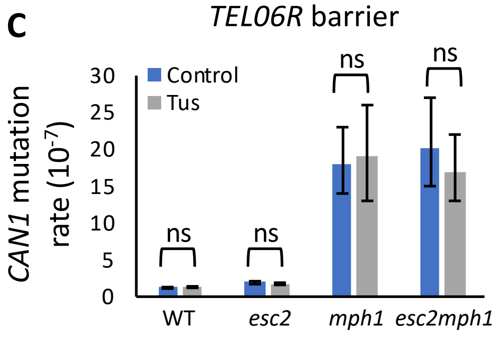

D

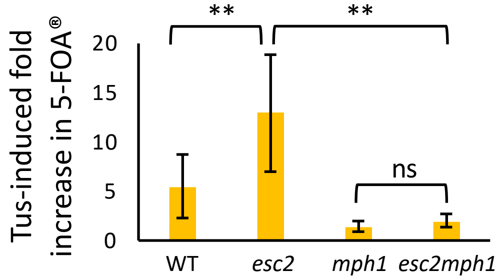

E

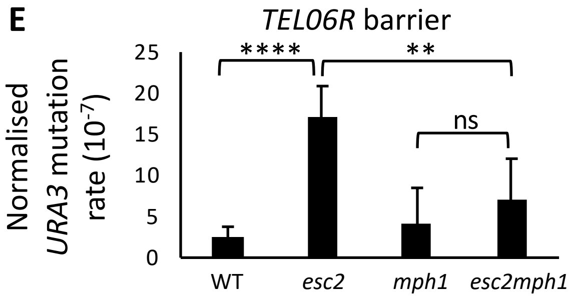

F

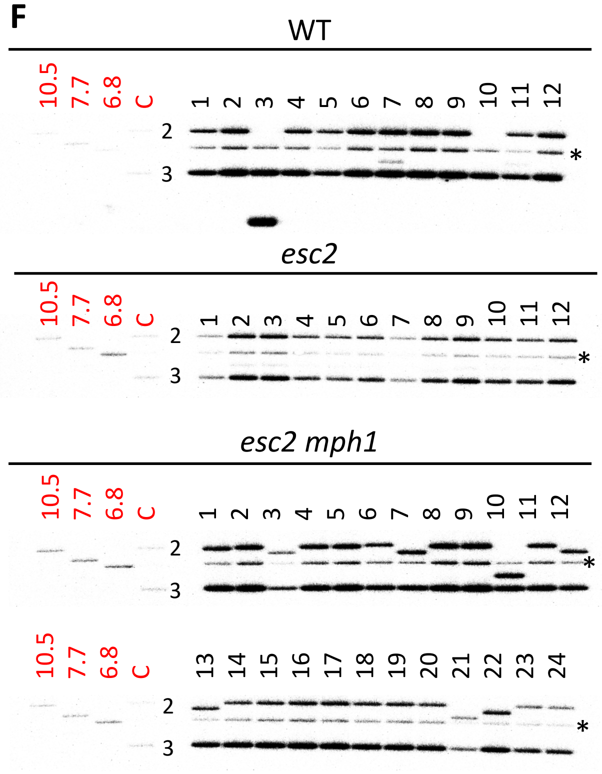

Supplementary Figure 4. Mutation type analysis. **(A)** From the same experiment shown in Figure 4B, *AfeI-SalI-EcoNI* fragments were analysed by 1DGE using probe 1 (left) or probe 2 (right), as described in Figure 4A. WT clones are shown at the top and *esc2* clones at the bottom. Markers and fragments detected are as per Figure 4B. The asterisk denotes a non-specific band detected in all samples with probe 2. **(B)** Diagram showing the sequence context surrounding the position of sequence loss at the most common truncation seen in *esc2* mutants. Data were obtained by telomere tailing and DNA sequencing. The sequence in black is retained, whereas the sequence in red is lost. **(C)** Induction of the Tus/*Ter* barrier does not affect *CAN1* mutation rates in *mph1* or *esc2 mph1* strains. *CAN1* mutation rates were measured in strains harbouring *TEL06R* Tus/*Ter* barrier. Data were analysed as described in Figure 1C. **(D)** Tus-induced fold increase in *URA3* mutation rate is plotted for the indicated strains. Error bars represent standard deviation from three independent experiments. Statistical analysis of differences was performed using an unpaired t-test and significance is indicated when $p<0.05$. **(E)** The normalised *URA3* mutation rate at *TEL06R* in cells expressing Tus was calculated after subtraction of the background level (in the control). Error bars represent standard deviation from three independent experiments. Statistical analysis of differences was performed using an unpaired t-test and significance is indicated when $p<0.05$. **(F)** From the same experiment as shown in Figure 6C, *AfeI-SalI-EcoNI* fragments were analysed by 1DGE using probe 2 (as shown in Figure 4A). WT clones are shown in the top panel, *esc2* clones in the middle panel, and *esc2 mph1* clones in the bottom two panels. Markers and fragments detected are as per Figure 4B. The asterisk denotes a non-specific band detected in all samples with probe 2.

Supplementary Figure 5

A

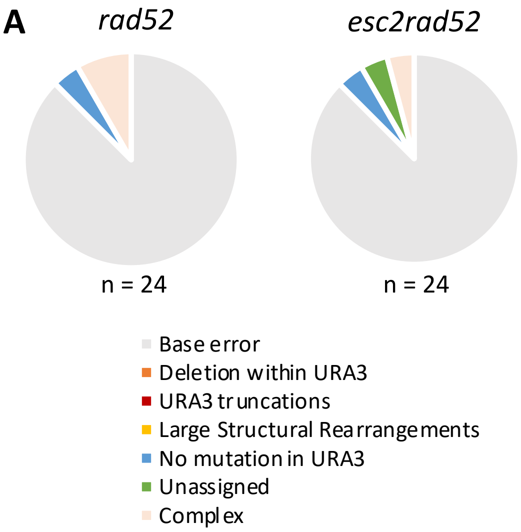

	rad52		***esc2rad52***	
	**n**	**Specific Mutation Rate (x10^{-7})**	**n**	**Specific Mutation Rate (x10^{-7})**
Base error	21	17.5	21	20
Deletion within URA3	0	0	0	0
URA3 Truncation	0	0	0	0
LSR	0	0	0	0
No Mutation in URA3	1	0.8	1	1
Unassigned	0	0	1	1
Complex	2	1.7	1	1
TOTAL	24	20	24	23

B

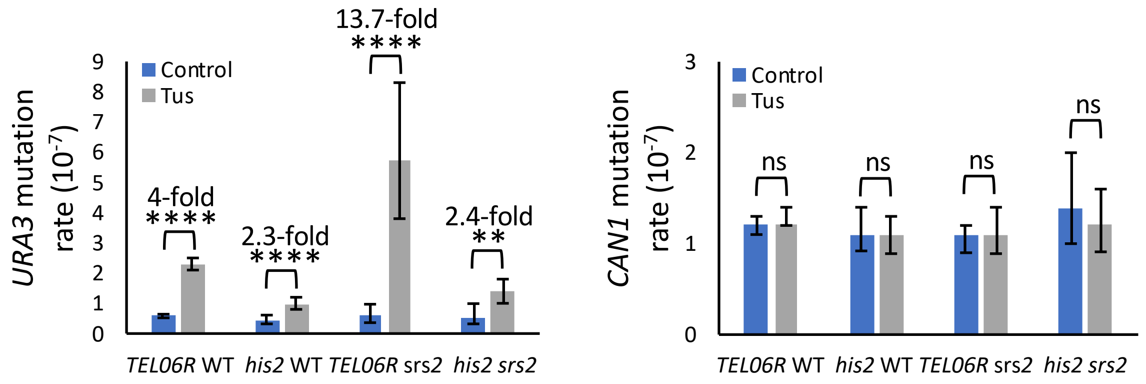

C

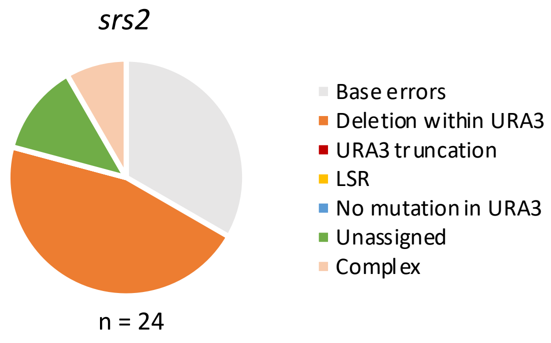

	srs2	
	**n**	**Specific Mutation Rate (x10^{-7})**
Base error	8	1.9
Deletion within URA3	11	2.6
URA3 Truncation	0	0.0
LSR	0	0.0
No Mutation in URA3	0	0.0
Unassigned	3	0.7
Complex	2	0.5
TOTAL	24	5.7

Supplementary Figure 5. Dysregulated HR at the *TEL06R* Tus/*Ter* barrier. **(A)** Mutation types were identified at the *TEL06R* barrier in the *rad52* (left) and *esc2rad52* (middle) mutant. The pie charts indicate the relative proportions of mutation types, as indicated in the key below. Data were obtained by DNA sequencing. The specific mutation rate for individual types of mutations was calculated (right). n = number of times a specific mutation type was observed. Colour coding corresponds to that in the pie chart. **(B)** Effect of *SRS2* gene deletion on *URA3* (left) and *CAN1* (right) mutagenesis at the *TEL06R* and *his2* Tus/*Ter* barrier was tested. Data were analysed as described in Figure 1C. **(C)** Mutation types were identified at the *TEL06R* barrier in the *srs2* mutant. The pie chart indicates the relative proportions of mutation types, as indicated in the key to the right. Data were obtained by DNA sequencing. Specific mutation rates for individual types of mutations are indicated. n = number of times a specific mutation type was observed. Colour coding corresponds to that in the pie chart.
